# Lymphangiome kystique du cordon spermatique: à propos d’un cas

**DOI:** 10.11604/pamj.2018.31.191.16709

**Published:** 2018-11-19

**Authors:** Mohamed Amine Oukhouya

**Affiliations:** 1 Centre Hospitalier Universitaire Hassan II, University Sidi Mohamed Ben Abdellah, Department of Pediatric Surgery, Fès, Morocco

**Keywords:** Cystic lymphangioma, infant, spermatic cord, Cystic lymphangioma, infant, spermatic cord

## Abstract

We here report the case of a 1-year old infant admitted to Hospital with left inguinoscrotal swelling evolving over the last 4 months, gradually increasing in volume and becoming painful. Clinical examination showed conscious, afebrile patient with stable hemodynamic and respiratory status but with left, soft, inguinoscrotal, transilluminable swelling painful to palpation. Inguinal doppler echocardiogram objectified 30 mm multiloculated mass. Abdominopelvic CT scan showed cystic lymphangioma: (A) shows the clinical image of the swelling; (B) shows the abdominopelvic CT scan image of a cystic lymphangioma; (C) shows the lymphangioma after exploration via the inguinal route which was in close contact with the spermatic cord and reaching the scrotum; resection of the cyst was performed after careful dissection, while preserving the noble elements; (D) shows patient’s outcome after 3 months. Anatomo-pathologic study confirmed the diagnosis of cystic lymphangioma. After 6-months’ follow-up contralateral swelling developed.

## Image en médecine

Nous rapportons le cas d'un nourrisson âgé de 1 an, admis à notre formation pour prise en charge d'une tuméfaction inguino-scrotale gauche évoluant depuis 4 mois et augmentant progressivement de volume et devenant douloureuse. L'examen clinique a trouvé un enfant conscient, stable sur le plan hémodynamique et respiratoire, apyrétique; avec une tuméfaction inguino-scrotale gauche molle transilluminable douloureuse à la palpation. Une échodoppler inguinale a objectivé une masse multiloculée de 30 mm. Une TDM abdomino-pelvienne a été en faveur d'un lymphangiome kystique (A) objective l'image clinique de la tuméfaction; (B) objective sur une TDM abdomino-pelvienne l'image en faveur d'un lymphangiome kystique; (C) objective l'aspect du lympangiome après exploration par voie inguinale qui était en contact intime avec le cordon spermatique et arrivant jusqu'au scrotum; on a opté pour la résection du kyste après dissection minutieuse tout en préservant les éléments nobles. (C) montre l'état final après 3 mois. La nature du kyste a été confirmée par l'étude anatomopathologique. Le recul est actuellement de 6 mois; on a remarqué l'apparition de la tuméfaction au niveau controlatéral.

**Figure 1:**
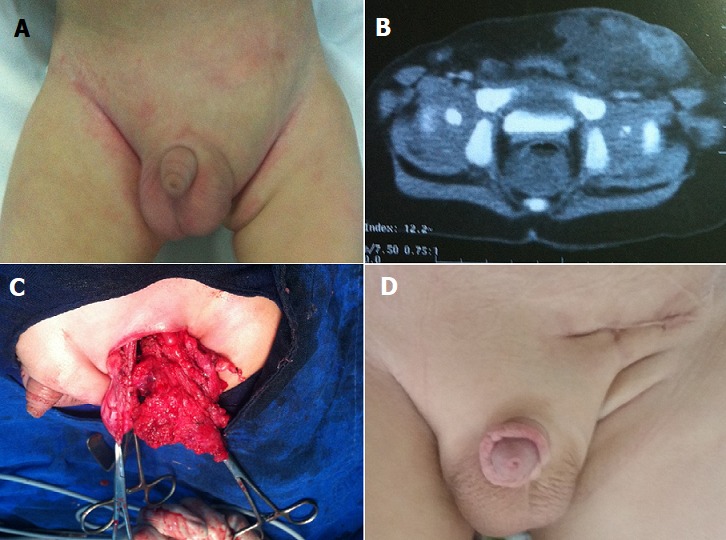
A) image clinique de la tuméfaction; B) TDM abdomino-pelvienne: image en faveur d’un lymphangiome kystique; C) aspect du lympangiome après exploration par voie inguinale qui était en contact intime avec le cordon spermatique et arrivant jusqu’au scrotum; D) état final après 3 mois

